# A Human Clinical and Histomorphometrical Study on Different Resorbable and Non-Resorbable Bone Substitutes Used in Post-Extractive Sites. Preliminary Results

**DOI:** 10.3390/ma12152408

**Published:** 2019-07-28

**Authors:** Ilaria De Tullio, Sergio Caputi, Giorgio Perfetti, Luan Mavriqi, Daniel Wismeijer, Tonino Traini

**Affiliations:** 1 Department of Medical, Oral and Biotechnological Sciences, University of Chieti-Pescara, 66100 Chieti, Italy; 2 Department of Oral Surgery and Implantology, University “ALDENT” of Tirana, 1023 Tirana, Albania; 3 Department of Oral Implantology and Prosthetic Dentistry, Academic Centre for Dentistry Amsterdam (ACTA), University of Amsterdam and Vrije University Amsterdam, 1081 Amsterdam, The Netherlands

**Keywords:** socket preservation, bone regeneration, Calcium Sulphate, Hydroxyapatite, biomaterials, bone substitutes, bone volume

## Abstract

Background: The healing of sockets following teeth extraction results in a marked reduction of the height and width of the ridge. This in vivo study aims to assess and compare the efficacy of calcium sulphate (CS) and sintered nano-hydroxyapatite (NHA) in postextraction sockets. Materials and Methods: 10 subjects were enrolled for single or multiple tooth extraction and implant placement. Each site was randomly assigned to one of four groups and filled with CS, NHA, a combination of CS and NHA, or left to normal healing. After five months tissue samples were harvested from the extraction sites and prepared for histological investigations. Results: Histomorphometric analysis showed that the average percentages of vital bone was 13.56% ± 13.08% for CS, 17.84% ± 7.32% for NHA, 58.72% ± 8.77% for CS + NHA%, and 80.68% ± 21.8% for the controls; for the connective tissue the results were 33.25% ± 35.75% for CS, 55.88% ± 21.86% for NHA, 17.34% ± 8.51% for CS + NHA, and 22.62% ± 0.52% for the controls; for residual biomaterial the results were 0.56% ± 0.52% for CS group, 21.97% ± 0.79% for NHA, and 47.54% ± 20.13% for CS + NHA. *Conclusions*: Both biomaterials led to bone tissue formation after five months of healing. The combination of the biomaterials presented a better behavior when compared to the individual application.

## 1. Introduction

The success rate of implant supported restoration can be directly correlated to the quantity and quality of bone around dental implants [1]. When insufficient bone volume is present, the implant insertion could be problematic: The alveolar process is a tooth-dependent structure that forms during tooth eruption and undergoes resorption after single or multiple extractions [2,3,4,5]. Height and width decrease of the ridge volume and subsequent deficiency of acceptable bone volume for optimal implant placement are common situations following tooth extraction [6]. 

Furthermore, several studies have demonstrated that the healing process after tooth removal resulted in marked alteration of the vertical and horizontal dimensions of the alveolar process [2,3,4,7].

Bone loss following tooth removal is more marked in the first six months and Lang et al. in 2012 demonstrated in their systematic review that the alveolar process experiences a mean horizontal resorption in width of 3.8 mm and a mean vertical resorption in height of 1.24 mm during that period.

Alveolar ridge preservation (also referred to as “socket preservation”) was defined as any technique designed to counteract, or even eliminate, the undesirable effects of post extraction resorption; to preserve the soft and hard tissue outlines of the ridge; to stimulate bone formation within the socket; and to simplify implant surgical procedures in a prosthetically determined position [8].

Thus, socket preservation has been revealed to change these modelling events, and so, partly counteracts the peripheral ridge contraction that follows tooth extraction [9]. This, according to the Osteology Consensus Report of 2012, based on the systematic review by Vignoletti et al. 2012, allows the preservation of the existing soft and hard tissue volume. This enhances the functional and aesthetic outcomes and can simplify the subsequent implant placement procedures [10]. 

This is in accordance with the conclusions of 4th EAO Consensus Conference in 2015, which stated that alveolar ridge preservation is a valid therapeutic concept, as it notably reduces the ridge resorption following the tooth removal, therefore improving the probability of implant insertion in a prosthetically guided position [11]. 

In attempts to counteract the alveolar ridge resorption following tooth removal, numerous graft materials including autogenous, allogeneic, xenogeneic, and alloplastic bone grafts have been used [9]. By definition, an ideal biomaterial should be osteoconductive, osteoinductive, osteogenic [12], economic, easy to handle, resorbable [13,14], and radiopaque, in order to be visible on x-ray. Among these properties, resorption is significant for obtaining tissue regeneration with “restitutio ad integrum” instead of a “tissue repair”: The ideal bone graft biomaterial should be a provisional template to encourage cell migration from the borders of the grafted area, thus promoting bone regeneration [15]. 

Hence, although different biomaterials with natural or synthetic origin have been used in augmentation bone procedures, as of yet there is no ideal bone substitute available [12]. 

Furthermore, during the 4th EAO Consensus Conference in 2015 it was stated that there is also a clear need for further research to establish which biomaterial is to be preferred [11].

In order to completely assess the healing process, bone graft materials should be evaluated histologically [16]. 

The aim of the present human experiment was to assess and compare the in vivo efficacy of calcium sulphate, chosen as a resorbable bone grafting material, and hydroxyapatite, chosen as a nonresorbable bone substitute, used individually or in combination to fill postextraction sockets.

Calcium sulphate is an easy to use and economic biomaterial, which is often used in dentistry. In the literature, histologic studies in animals showed calcium phosphate particles surrounding calcium sulphate during graft remodeling [17]. Histological studies in humans showed that after six months all calcium sulphate particles are substituted by calcium phosphate [18]. Many authors assess that this process is completed in the first 4–10 weeks of bone healing and is dependedent upon the site’s vascularization [19].

Hydroxyapatite is a widespread and available material synthetized in laboratory environments with osteoconductive properties. The improvement of the industry’s technologies has created a new synthetic nanohydroxyapatite with porosities in the order of nanometers, which seems to have osteoinductive properties: The porous hydroxyapatite ceramic granules are able to induce bone regeneration in muscle tissue (ectopic sites) and remain in the site for years [12,20,21,22].

## 2. Materials and Methods

### 2.1. Study Design and Population

The present study was performed in accordance with the ethical principles expressed in the declaration of Helsinki as modified in Fortaleza (2013) for research on human subjects. The ethical committee of the University Aldent of Tirana (Albania) approved the study protocol (Protocol number: 2).

All procedures referred to in this study were performed at Brianza Dent clinic in Tirana (Albania), which is affiliated with the University Aldent of Tirana (Albania).

Patients signed a written informed consent form before participating in the study: They were carefully informed about the study protocol, the treatment and its options, the benefits, and the possible dangers. 

This human clinical trial tested the null hypothesis: There is no difference in new bone formation between nanohydroxyapatite (NHA) bone grafts (test group), calcium sulphate (CS) bone grafts (test group), NHA and CS mixed bone grafts (test group), and no grafts (control group), in postextraction sockets.

Ten subjects were enrolled for single tooth or multiple teeth extraction and implant-supported restoration, and both mandibular and maxillary sites were included.

The patients were male or female between 24 and 63 years old, in good systemic condition fitting the inclusion criteria (inclusion criteria were: ASA I or ASA II male or female between 30 and 70 years old, and requiring tooth extraction and bone regeneration procedure to insert delayed implant). Exclusion criteria for this experiment were patients with systemic risk factors that could prejudice the healing process of surgical wound such as: Diabetes, smoking habits, coagulation diseases, pregnant women, immunocompromised patients, patients with radio or chemotherapy history in last 6 months, and antiplatelet drugs administration before surgery.

Patients requiring a single tooth extraction, or each tooth site for patients who were included with multiple tooth extractions, were divided into 4 groups: 3 test groups and one control group:

1. Calcium Sulphate Test group (CS);
2. Nanohydroxyapatite Test group (NHA);
3. Calcium Sulphate plus Nanohydroxyapatite Test group (CS + NHA);
4. No biomaterial: Control group (Ctr).

Each site was randomly assigned to one of the three test groups or to the control group: a computer-generated table, distributing each extraction site into four groups (three tests and one control), was prepared using a balanced, randomly permuted block approach (http://www.randomization.com/).

In the case of multiple extraction sites, these had to be allocated in different quadrants so the allocated treatment could not influence a treatment the neighboring site. 

Numbered, opaque, sealed envelopes held the randomization codes and they were opened before starting surgical procedures.

### 2.2. Surgical Procedures

After administering a local anaesthetic (articaine 4% with epinephrine 1:100.000, Artin, Omnia, Italy), tooth extraction was performed with a flapless approach by using elevators and forceps. Extreme care was taken to not damage the socket’s walls. 

**Figure 1:**
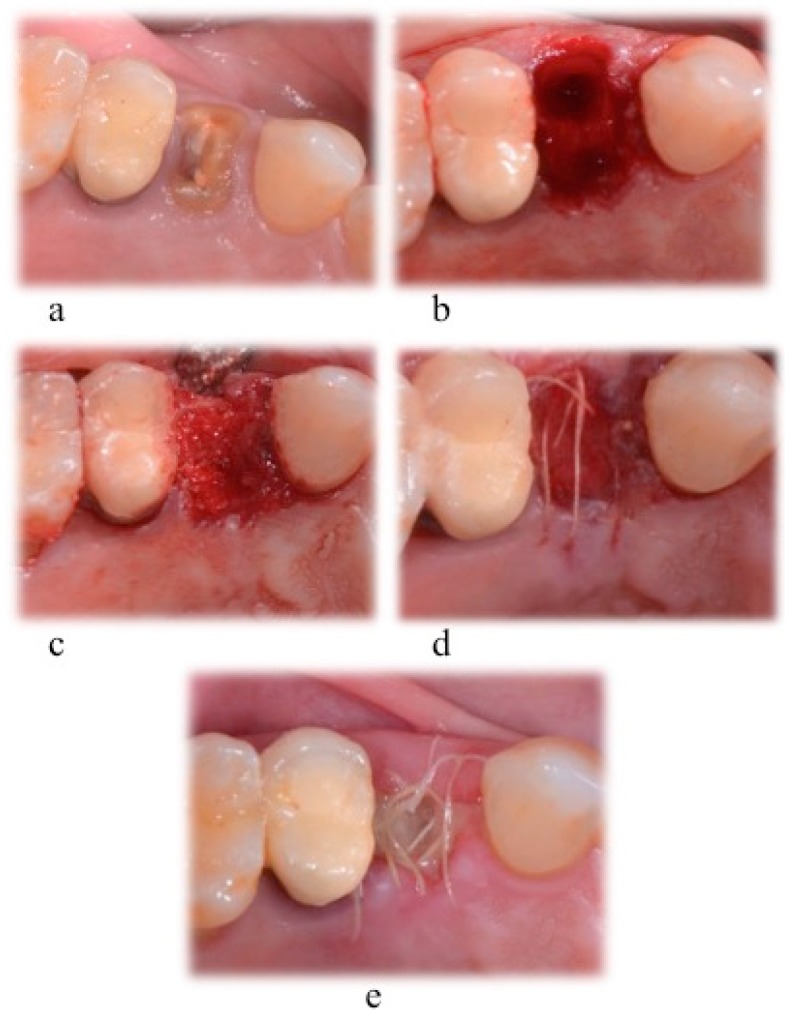
Tooth extraction and socket preservation. Surgical phases for: (**a**) Local anesthesia; (**b**) extraction socket; (**c**) socket filled with biomaterial; (**d**) suture with 4–0 vycril suture; (**e**) 1 week healing.

In the three test groups, the socket was filled with CS (G170 Surgiplaster, Ghimas, Bologna, Italy), with sintered NHA (Fisiograft Bone Granular, Ghimas, Bologna, Italy), or with a combination of 50% CS and 50% of NHA. The coronal part of the hard tissue wound was covered with a pressed fibrin sponge used as a membrane. In the control group, the socket space was left to normal healing, starting by the filling of the blood clot. The wound was sutured with a buccal-lingual/palatal cross suture using a 4/0 vycril suture (Figure 1).

Antibiotic prophylaxis with a broad-spectrum antibiotic was prescribed starting just before the surgery and then continued for 6 days (amoxicillin 2 g one hour prior to surgery and 1 g twice a day for 6 days after surgery, or clarithromycin 250 mg three times a day in patients allergic to penicillins) [23]. When required, non-steroid anti-inflammatory drug (NSAID) was additionally prescribed (ibuprofen 600 mg).

**Figure 2:**
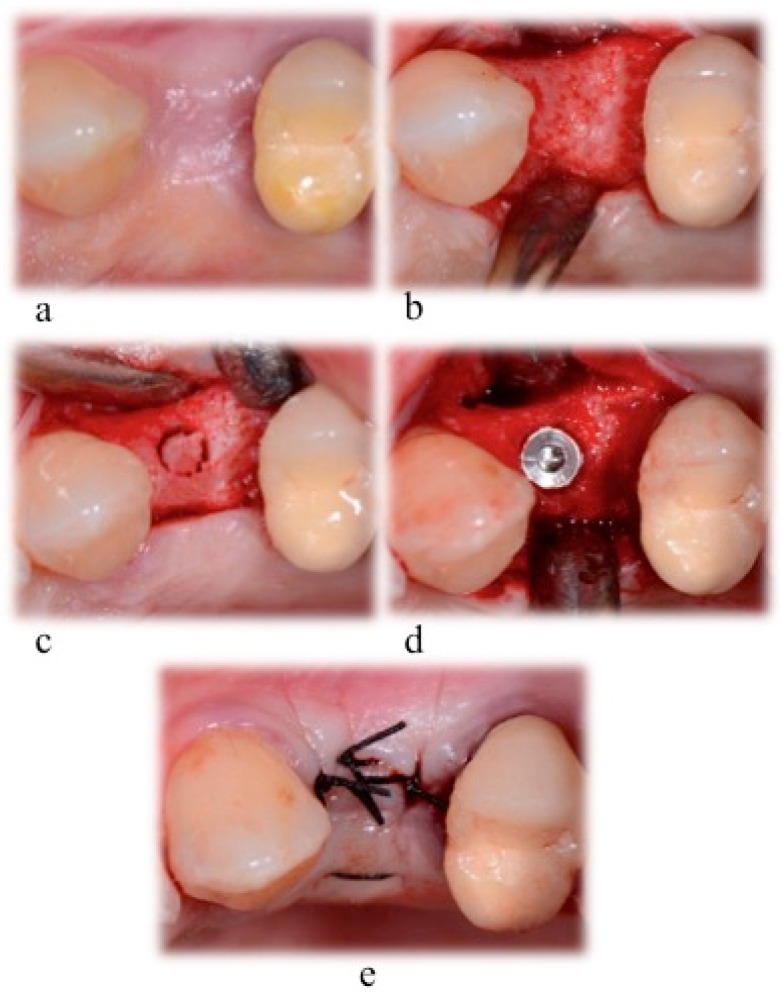
Surgical phases for implant bed preparation and sample harvesting, (**a**) after the infiltration of local anesthesia; (**b**) soft tissue incision and bone exposure; (**c**) hard tissue sample collecting phase; (**d**) implant insertion; (**e**) suture with a 4–0 silk suture.

Expected healing times were between 4 and 6 months. After 5 months, a hard tissue sample was harvested from each extraction site: After a full thickness flap elevation, as part of the implant bed preparation, a trephine bur with an inner diameter of at least 2 mm (2.8 mm of outer diameter and 2 mm of inner diameter; or 3.5 mm of outer diameter and 3 mm of inner diameter) was used to remove a block of hard tissue (Figure 2). After the implant placement procedure, a 4/0 pluri-filament nonresorbable suture was used to close the surgical wound (horizontal mattress and simple “o” sutures). 

**Figure 3:**
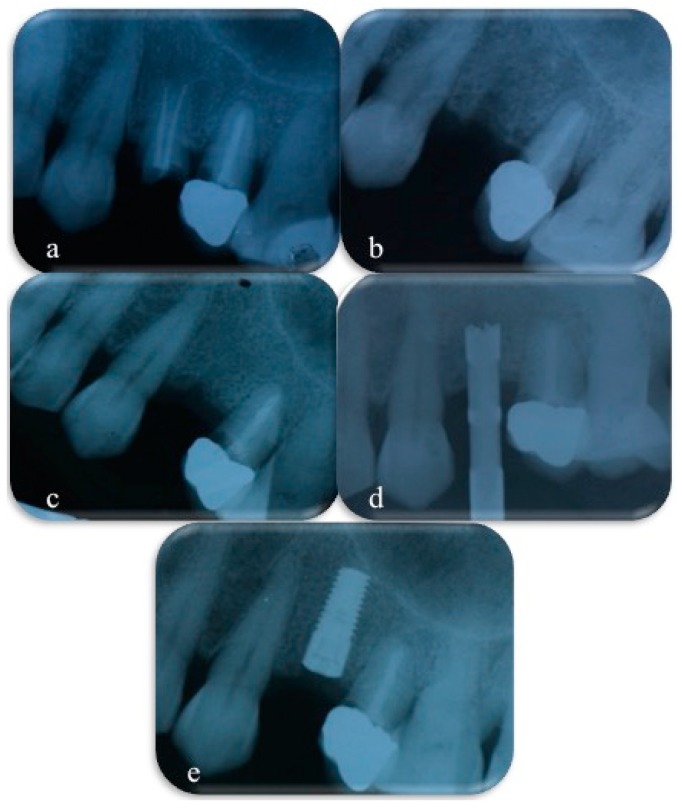
X-rays collected before tooth extraction (**a**), immediately after tooth extraction and socket preservation procedures (**b**), after 4 months of healing (**c**), during bone sample collecting with a trephine bur (**d**), immediately after implant placement (**e**).

The specimen collection phase was extensively documented in order to collect all possible data useful to the examiner; data included patient ID, information about sample site, photographs, OPT, and intra-oral X-rays (Figure 3).

The biopsies, left inside the trephine burs, were rinsed for 40 s with a cold 5% glucose solution to remove blood while maintaining the correct osmolarity (278 mOsm/L). The hard tissue samples were then immediately stored in a case with an adequate volume of 10% formalin solution (at least ten times the sample’s volume) and buffered at pH 7.2. To prevent transport damage, each specimen was stored in a separate container with cotton added. Finally, each sample’s case was labelled with patient ID and tooth number. During the processing stage patient ID and tooth number were provided with a unique numerical code.

To prepare the samples for histological investigation, a specific protocol was followed. The hard tissue biopsies were washed twice with phosphate buffered saline solution and dehydrated with alcohol in a graded series and at 4 °C for seven days. After obtaining the complete dehydration by using absolute alcohol for two additional days, the specimens were infiltrated in a 20% solution of resin (LR White EM, TAAB Laboratories Equipment Ltd., Aldermaston, UK) and alcohol for ten days. The resin/alcohol ratio of the solution was changed to 50% for another 7 days and then the specimens were completely infiltrated in 100% resin (two changes). This phase was conducted using a vacuum chamber for 15 additional days, or longer, until the hard tissue biopsies had become transparent. At the end of this stage, the samples were removed from the trephine bur easily, using a custom-made plunger (thanks to the shrinkage consequent to the phases of dehydration and resin embedding), oriented, put in a small Eppendorf with resin, and polymerized at 60 °C for 12 h. Then, the biopsies were cut along their longer axis at about 50 μm, using a high-precision diamond disc (TT System, TMA2, Grottammare, Italy). The slices were reduced in thickness to about 30 ± 10 μm by grinding with a series of polishing discs from 400 to 1200 grits under cold water and then by a final polish with 0.3 μm alumina in a microgrinding system (TT System, TMA2, Grottammare, Italy) [22]. 

The sections were stained with Toluidine Blue and Azure II and counterstained with acid fuchsine and mounted on glass slides for observation. The microscopic analysis was conducted by connecting a high-resolution digital camera (FinePix S2 Pro, Fuji Photo Film, Tokyo, Japan) to a transmitted bright-field microscope (BX 51, Olympus America, Waltham, MA, USA) and to a bright-field/circularly polarized light (CPL) microscope (Axiolab, Zeiss, Germany). 

A quarter-wave retarder and two polarizing filters were combined to generate CPL: The quarter-wave retarder was positioned just below the filter called analyzer, one circular polarized filter was positioned directly beneath the glass, below the sample, and the other filter (the one called analyzer) was placed on the superior end of the objective, above the sample. 

In CPL microscopy, all the fibers are lightened near equally, irrespective of their direction. The lack of specificity for direction in enlightening collagen fibers makes CPL perfect for image processing techniques used to count their amount and their orientation. In order to have different levels of resolution to analyze the collagen fibers’ direction, the analyzer was turned at −5° and the quarter-wave retarder at +5° or vice versa.

**Figure 4:**
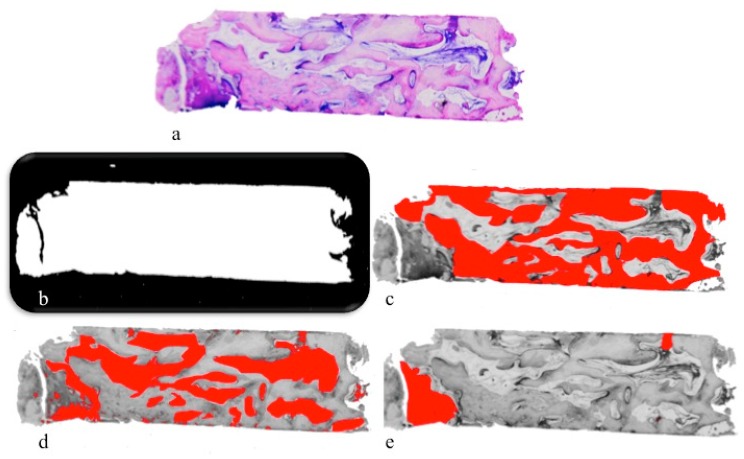
The sections of the harvested biopsies (**a**), analyzed for: Mean surface of the specimen (**b**); newly formed bone area (**c**); connective tissue area (**d**); residual graft particles area (**e**).

The digital photomicrographs were used to perform histomorphometric analysis, measuring the following parameters: (1) Amount of tissue collected with the biopsies over the obtained sections (area of samples); (2) amount of vital bone as absolute value (mm^2^) and as relative value (vital bone area/total sample area ×100); (3) marrow space (connective tissue) as absolute value (mm^2^) and as relative value (connective tissue area/total sample area ×100); (4) residual graft particles as absolute value (mm^2^) and as relative value (biomaterial area/total sample area ×100). A histometric software set able to take and save pictures was used (Image-Pro Plus 6.0, Media Cybernetics Inc., Rockville, MD, USA) (Figure 4). For each experimental image, the software was calibrated using the inner diameter of the trephine bur to calculate the distance.

### 2.3. Outcomes

This study evaluated the quality of the developed tissue: (1) New bone formation (as a percentage of newly developed bone area over total measured area) and its architecture, (2) residual graft particles and their distribution (as a percentage of graft particles area over total measured area), and (3) marrow bone and vessels (as a percentage of connective tissue area over total measured area).

### 2.4. Statistical Analysis

Statistical analysis was performed for the following measurements: (1) Amount of tissue collected with the biopsies over the total sample area obtained with the biopsies; (2) amount of vital bone as absolute value (mm^2^) and as relative value (vital bone area/total sample area ×100); (3) marrow space (connective tissue) as absolute value (mm^2^) and as relative value (connective tissue area/total sample area ×100); (4) residual grafting material as absolute value (mm^2^) and as relative value (biomaterial area/total sample area ×100).

Data expressed as mean values (±SD) were analyzed by a computerized statistical software (GraphPad Prism6 by Software MacKiev©, 1994-2014 GraphPad Software, Inc., San Diego, CA, USA) using the one-way analysis of variance (ANOVA) followed by Tukey post hoc test. A *p*-value <0.05 was set as statistically significant.

## 3. Results

### 3.1. Clinical Results

In total, 10 patients (aged 47.47 ± 13.43 years, range: 24–63 years, five females, five males) were enrolled and randomly assigned to one of the three test groups or to the control one. Six patients contributed with one single site and four patients with multiple sites, thus when one patient had more than one site, each site was considered as a new unit. Each of the 16 sites was grafted with NHA, CS, NHA and CS together, or left to normal healing. 

Eight patients were nonsmokers and two smokers. The principal standard patient characteristics are briefly reported in Table 1.

Reasons for tooth extraction were severe decay with or without periapical lesion, advanced periodontal disease with at least two walls still present, or for prosthetic reasons (patient referred for a complete all-on-four rehabilitation).

The NHA test group included one canine, one premolar, and two molar sites; three from the lower jaw and one from the upper jaw. The CS test group included one canine, two premolar, and one molar site; three from the lower jaw and one from the upper jaw. The CS + NHA test group included one incisor, one premolar, and two molar sites; two from the lower jaw and two from the upper jaw. The control group included one premolar, and three molar sites; one from the lower jaw and three from the upper jaw. In the CS test group, biopsies were harvested from one canine and two premolar sites; two from the lower jaw and one from the upper jaw. In the NHA test group, biopsies were harvested from one premolar, and two molar sites; two from the lower jaw and one from the upper jaw. In the CS + NHA test group, biopsies were harvested from one incisor, one premolar, and one molar site; two from the lower jaw and one from the upper jaw. In the control group, biopsies were harvested from one premolar, and two molar sites; one from the lower jaw and two from the upper jaw.

No intraoperative complications were documented and the healing phase following tooth extractions, or tooth extraction followed by socket preservation, was uneventful in all patients.

Five months of healing after tooth extractions and grafting procedures, an obviously noninflamed keratinized gingiva covered the coronal part of all test and control sites; twelve bone specimens were collected from eight patients (three biopsies in CS test sites, three biopsies in NHA test sites, three biopsies in CS + NHA test sites, and three in control sites). 

### 3.2. Histological Results

**Figure 5:**
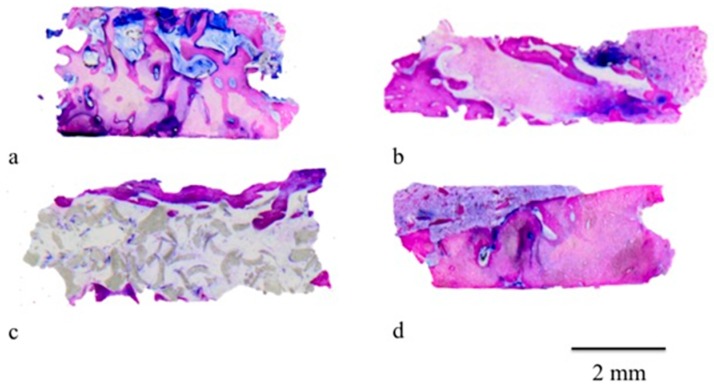
The sections of the harvested biopsies: Calcium sulphate plus nanohydroxyapatite (CS + NHA) groups (**a**); calcium sulphate (CS) groups (**b**); nanohydroxyapatite (NHA) group (**c**); control group (**d**). Original magnification 12×.

Nongrafted sites: A layer of solid cortical bone of variable width coated the marginal portion of the specimens from the nonaugmented extraction sites (Figure 5). The middle and apical portions were comprised of bone with few bone marrow spaces (Figure 5). The total area of the hard tissue biopsies consisted of newly formed bones embedded in bone marrow spaces. 

**Figure 6:**
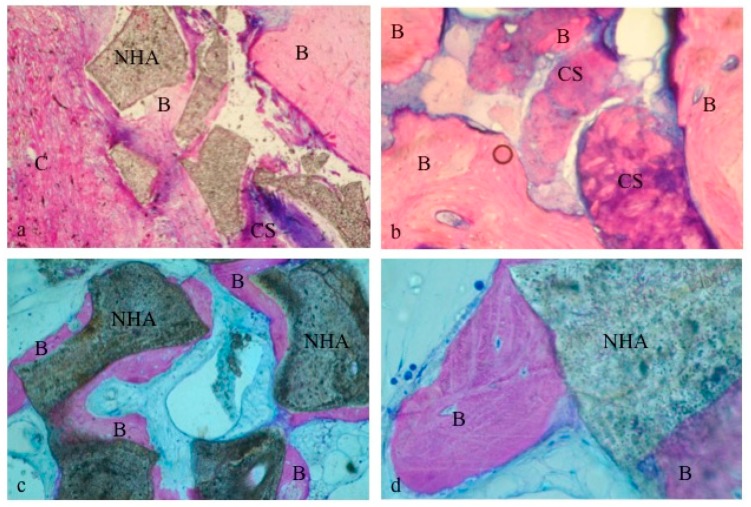
Biomaterial particles appeared to be surrounded by new bone: CS + NHA group (**a**); CS group (**b**); NHA group (**c**,**d**). B: bone; C: connective tissue; CS: Calcium Sulphate; NHA: sintered nanohydroxyapatite. Original magnification 40× (**a**,**c**) and 100× (**b**,**d**).

Grafted sites: Both the CS and the CS + NHA test sites presented a layer of mineralized bone, sealing the coronal part of the biopsies. A lesser amount of new bone was noted in the NHA test sites (Figure 5). The lateral walls of the specimens and the more central portions of the CS and CS + NHA biopsies consisted of struts of newly developed bone and several marrow areas. On the surface of the residual biomaterial particles, newly formed bone was present (Figure 5 and Figure 6). However, some NHA particles were apparently not associated with struts of new bone formation and vice versa surrounded by connective tissue (Figure 5).

After five months of healing, both the biomaterials used in this experiment showed a good level of “osseointegration”. Graft residual particles were distinguishable from the other components of the regenerated tissue and seemed to be fused by bridges of newly developed bone (Figure 6). This feature indicates considerable osteointegrative and osteoconductive properties of the external surface of the biomaterials. Furthermore, bone surrounding the grafted particles was characterized by osteocytes surrounded by the mineralized bone matrix (Figure 6).

**Figure 7:**
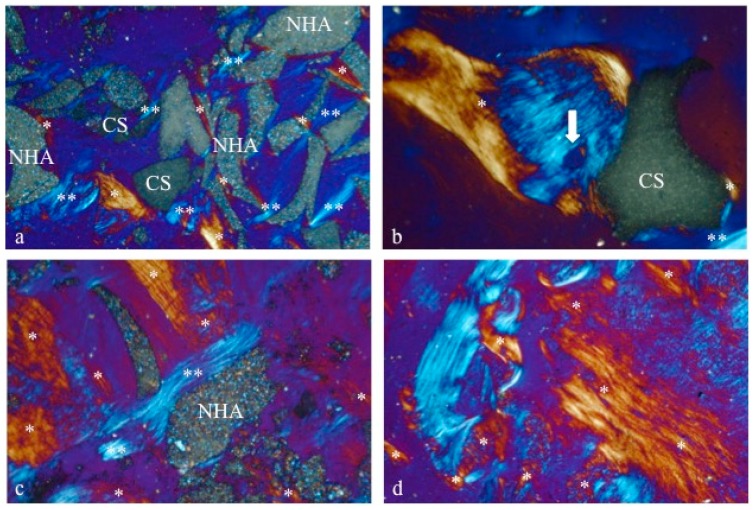
Under circularly polarized light (CPL), the differences in the microstructure and relationship between the two biomaterials and the newly formed bone could be observed. The new bone, surrounding some CS particles, showed vessels (white arrow in picture b indicates a Volkman Canal). Moreover, the intimate contact between the new bone (*) and the biomaterials (CS and NHA) revealed clearly the osteoconduction properties (**a**–**d**). CS: Calcium sulphate; NHA: Sintered nanohydroxyapatite; *: Newly formed bone; **: Desmoplastic connective tissue. Original magnification 100×.

Using a circularly polarized light microscope, newly formed bone seemed to surround both biomaterials’ particles (Figure 7), thus confirming the osteoconductive properties. Moreover, the collagen fibers’ orientation showed that some NHA particles were embedded in a desmoplastic connective tissue in samples obtained from postextraction sockets filled with a combination of NHA and CS (Figure 7a). In fact, CS resorption led to the formation of new bone after its first replacement by this connective tissue (Pettinicchio et al.). If on one hand, this process seems to retard new bone deposition, on the other hand, it increases bone vessels growth (Figure 7b).

This process could explain the presence of this tissue around some NHA particles (Figure 7c) after five months of healing: As NHA and CS biomaterials were used in combination, the space filled with CS resulted in it being replaced by the newly formed desmoplastic connective tissue (Figure 7c), which would subsequently be substituted with new bone (Figure 7d).

### 3.3. Histomorphometric Results

The histomorphometric measurements were performed using the histometric software package (Image-Pro Plus 6.0, Media Cybernetics Inc., Rockville, MD, USA) (Figure 4).

The sections of the harvested biopsies had a mean surface area of 24.62 ± 12.58 mm^2^ for CS group, 8.53 ± 0.85 mm^2^ for NHA group, 23.49 ± 7.85 mm^2^ for CS + NHA group, and 9.62 ± 1.81 mm^2^ for the control group. The differences among the groups were all not statistically significant (ANOVA with Tukey post hoc test; *p* > 0.05).

The bone area was 2.29 ± 1.12 mm^2^ for CS group, 1.48 ± 0.47 mm^2^ for NHA group, 13.34 ± 2.66 mm^2^ for CS + NHA group, and 7.5 ± 0.6 mm^2^ for the control group. The difference between the test groups and the control group was statistically significant (ANOVA with Tukey post hoc test; *p* < 0.05); the differences between CS + NHA test group and both the CS and the NHA test groups were statistically significant (ANOVA with Tukey post hoc test; *p* < 0.05); the difference between the NHA test group and CS test group was not statistically significant (ANOVA with Tukey post hoc test; *p* > 0.05).

The connective tissue area was 5.71 ± 3.25 mm^2^ for CS group, 4.89 ± 2.33 mm^2^ for NHA group, 3.91 ± 1.99 mm^2^ for CS + NHA group, and 2.17 ± 0.36 mm^2^ for the control group. The differences among the groups were all not statistically significant (ANOVA with Tukey post hoc test; *p* > 0.05).

The area occupied by residual grafting particles was 0.17 ± 0.14 mm^2^ for CS group, 1.87 ± 0.12 mm^2^ for NHA group, and 1.22 ± 0.82 mm^2^ for CS + NHA group. The differences between the NHA and CS + NHA test groups and the control group were statistically significant (ANOVA with Tukey post hoc test; *p* < 0.05), while the difference between the CS test group and the control group was not statistically significant (ANOVA with Tukey post hoc test; *p* > 0.05). The difference between the NHA and CS test groups was statistically significant (ANOVA with Tukey post hoc test; *p* < 0.05), while differences between CS + NHA test group and CS and NHA tests groups were both not statistically significant (ANOVA with Tukey post hoc test; *p* > 0.05).

**Figure 8:**
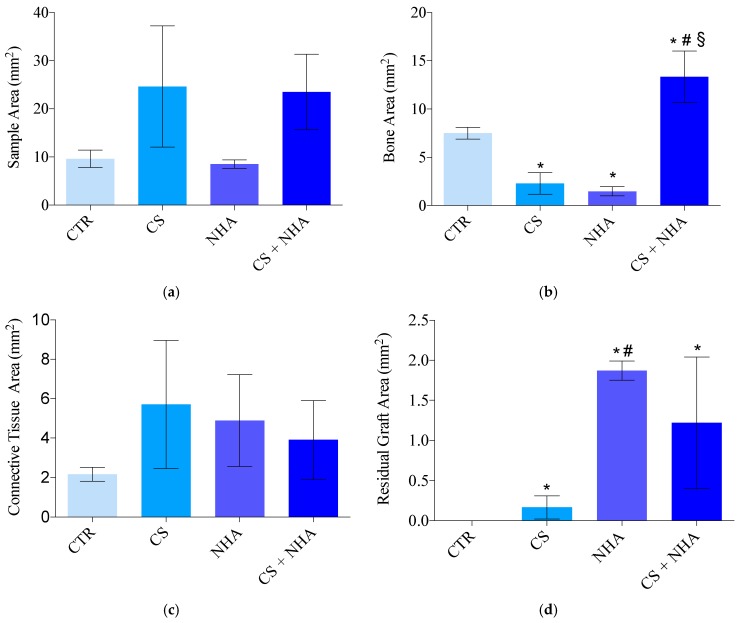
Sample area in mm^2^ (**a**), bone area in mm^2^ (**b**), connective tissue area in mm^2^ (**c**), residual graft area in mm^2^ (**d**). CTR: Control; CS: Calcium sulphate; NHA: Sintered nanohydroxyapatite; CS + NHA: Calcium sulphate plus sintered nanohydroxyapatite; *: Significantly different compared to the control group; #: Significantly different compared to the calcium sulphate group; §: Significantly different compared to the NHA group.

The results are reported in Table 2 and Figure 8.

The average percentage of vital bone was 13.56% ± 13.08% for CS group, 17.84% ± 7.32% for NHA group, 58.72% ± 8.77% for CS + NHA group, and 80.68% ± 21.8% for the control group. The difference between the CS + NHA test group and the control group was not statistically significant (ANOVA with Tukey post hoc test; *p* > 0.05), along with the difference between the NHA test group and the CS test group (ANOVA with Tukey post hoc test; *p* > 0.05). The differences between the CS + NHA test group and CS and NHA groups were statistically significant (ANOVA with Tukey post hoc test; *p* < 0.05), along with the differences between the NHA and the CS test groups and the control group (ANOVA with Tukey post hoc test; *p* < 0.05). 

The average percentage of connective tissue was 33.25% ± 35.75% for CS group, 55.88% ± 21.86% for NHA group, 17.34% ± 8.51% for CS + NHA group, and 22.62% ± 0.52% for the control group. The differences among all the groups were not statistically significant (ANOVA with Tukey post hoc test; *p* > 0.05).

The average percentage of residual grafting material 0.56% ± 0.52% for CS group, 21.97% ± 0.79% for NHA group, and 47.54% ± 20.13% for CS + NHA group. The difference between the CS test group and the control group was not statistically significant (ANOVA with Tukey post hoc test; *p* > 0.05); on the contrary, all the other differences among the biomaterials and between the biomaterials and the control group were statistically significant (ANOVA with Tukey post hoc test; *p* < 0.05). 

**Figure 9:**
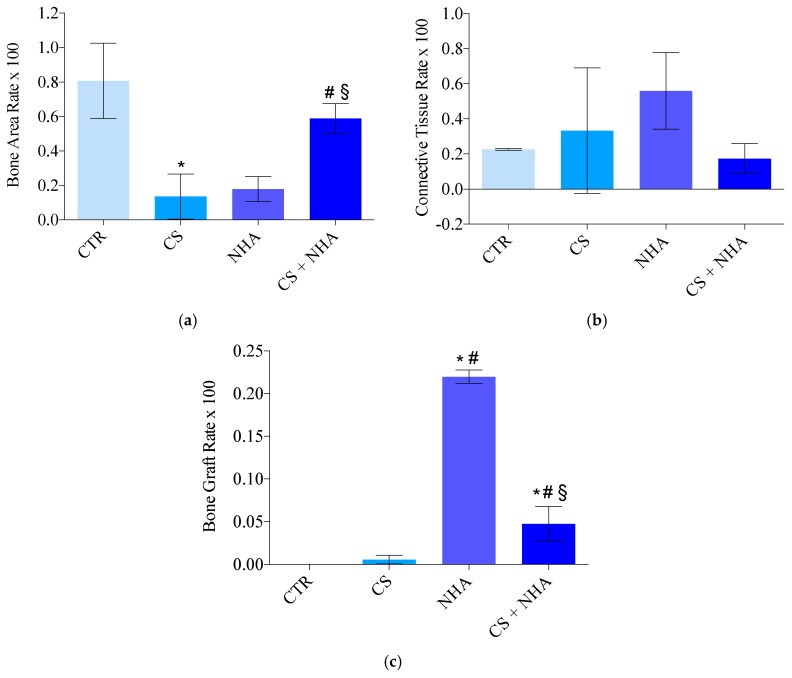
Histomorphometric measurements expressed in percentage: bone area rate (**a**), connective tissue rate (**b**), bone graft rate (**c**). CTR: Control; CS: Calcium sulphate; NHA: Sintered nanohydroxyapatite; CS + NHA: Calcium sulphate plus sintered nanohydroxyapatite; *: Significantly different compared to the control group; #: Significantly different compared to the calcium sulphate group; §: Significantly different compared to the NHA group.

Results are reported in Table 3 and Figure 9.

## 4. Discussion

The healing of sockets following tooth extraction was described as a sequence of events that starts from the establishment of a blood clot and its maturation. This goes on for several months until bone tissue formation, maturation, and remodeling [2,24,25,26,27] occur.

In particular, Cardaropoli, Araújo, and Lindhe in 2003 found in their investigation on dogs that starting from the third day, granulated tissue replaced the retained coagulum [23,28,29]. This seems to serve as a barrier from the bacteria in the oral cavity [30] and is displaced, after seven days, by a provisional matrix rich in blood vessels, collagen fibers, leukocytes, immature mesenchymal cells, and large areas of coagulative necrosis in the apical and central zones of the alveolar socket. After 14 days the coronal part of the alveolus is covered by an immature connective tissue under the epithelium layer, while the woven bone extends towards the center from the walls of the socket, made by old bone, thus showing that bone regeneration starts after two weeks of healing. Further investigations of the same study after 30 days, showed the start of newly formed bone remodeling and a socket almost filled with it [30]. After 60 and 90 days, a newly hard tissue bridge separated the epithelium from the socket, and most of the woven bone that filled the healing space was replaced by marrow bone: In specimens taken after 90 days, several areas showed lamellar bone and remodeling of the old walls of the socket. Finally, after 120 and 180 days of healing, collagen fibers from the lining mucosa reached the bone bridge, standing over layers of lamellar bone, and formed a periosteum like structure [30]. However, the phase of bone mineralization requires much more time and is also much less predictable [25].

These findings are in accordance with the clinical and histological observations of the present study: All the patients who underwent the second stage surgery showed a “cortical healing cap” on the postextraction sites, and in almost all the specimen collected after five months of healing the central portions, as well as the lateral ones, were made by struts of newly formed bone with several marrow spaces.

Even if all these events occurring after tooth extraction lead to healing with histological “restitution ad integrum” of bone architecture, bone healing after tooth removal usually results in hard and soft tissue remodeling and bone resorption [4,6,31,32]. 

Araújo and Lindhe in 2005 demonstrated in their experiment on dogs’ premolars that dimensional changes occurred during the first eight weeks after tooth removal. Bone resorption in the buccal and lingual/palatal aspect occurs because the bundle bone, after losing its function due to tooth extraction, is resorbed and then replaced by woven bone [6]. 

The socket bone wall’s alterations have been described to be more pronounced during the first six months of healing, involving more of the buccal bone than at the palatal/lingual aspect, and followed by a gradual modeling and remodeling of the residual bone: It is estimated that in the first six months as much as 60% of alveolar width and 40% of the alveolar height are lost [7]. 

These findings are of paramount importance in implant dentistry, as dental implants require an adequate amount of bone to be able to be inserted. The most important aim of bone regeneration in the implant practice is the creation of a suitable volume of tissue of good quality, in which to place dental implants so they can have a good long-lasting prognosis [22]. 

Function and aesthetics in implant therapy may be influenced by marginal crestal bone loss: Peri-implant bone loss can cause early implant failure and affect long-term peri-implant health and success [33]. 

Therefore, all the phases of implant dentistry should focus on minimizing bone loss, starting from the very beginning. Whenever the residual bone is insufficient to insert an implant, bone augmentation procedures could be performed to achieve long-term good functional and aesthetic results [1].

Paolantonio et al. in 2001 suggested placing implants in fresh extraction sockets in order to avoid ridge resorption, so preserving the original volume and shape of the ridge [34]. However, Botticelli, Berglundh and Lindhe in 2004 could not support this hypothesis with the findings reported from their clinical study [22]. The authors described that both the buccal and the lingual bone plates experienced marked resorption and remodeling during the first four months of healing after implant installation (buccal bone: 50%; lingual bone: 25%), at sites where implants had been inserted immediately after single tooth removal. These results were confirmed by an experiment by Araújo et al. in 2005 on dogs: They stated that immediate implant placement into a fresh extraction socket failed to counteract the remodeling of the ridge contours. 

Even though bone modeling and remodeling consequent to a tooth removal is not fully avoidable, the ridge preservation procedure allows for counteracting the bone loss after tooth extraction [31,35,36,37].

The Osteology Consensus Report of 2012, based on the systematic review of Vignoletti et al. in 2012, concluded that ridge preservation allows for the conservation of the remaining soft and hard tissue contour, a firm bone volume for aesthetic and functional outcomes, and can simplify the subsequent implant placement procedures [10,38].

Moreover, there is evidence that alveolar ridge preservation reduces the clinical need for further bone graft procedures during implant placement, even though there is still no indication on a time point to be preferred for implant bed preparation after this procedure [11]. 

Different bone grafting materials have been developed to regenerate residual bone but the association between the foreign grafting material’s presence in the living bone and the possible decrease of its mechanical competence is still not clear.

An ideal bone substitute material should guarantee a biologic scaffold, maintain volume, and induce the regeneration of vital bone and bone remodeling [16]. To completely evaluate the biological behavior of biomaterials and their involvement in the normal healing processes, histological and histomorphometrical evaluations are of primary interest [16].

The present research demonstrated differences with respect to the socket healing, five months after tooth removal, between sockets sites that underwent regenerative procedures by immediately refilling them with different biomaterials, and nongrafted sites. In particular, the bone area percentage of the nongrafted sites was significantly greater, if compared with the sites grafted with biomaterials alone (Figure 9). However, there were no statistical differences between the sites grafted with the combination of CS and NHA and the control sites, for bone area percentage (Figure 9). Moreover, there were no statistical differences among the groups, regarding the bone marrow spaces (expressed as connective tissue percentage in Figure 9). 

These results confirm that the combination of NHA, used as nonresorbable biomaterial, and CS, as a resorbable one, could be a valid option in socket preservation procedures. It results in the same proportion of newly formed bone as the sites left to normal healing, thus not interfering with the biological processes involved in bone healing. Moreover, even though the combination of biomaterials showed higher values of the residual particles rate compared with the control and the CS groups, it had lower residual graft particles rate than NHA used alone (Figure 9). In fact, the biomaterial that showed the minimum quantity of residual particles rate was the CS (Figure 9), and this is in agreement with the findings of previous studies. It was showed that CS quickly resorbs leaving a calcium phosphate pattern which stimulates osteogenic activity and mimics the bone’s mineral phase, showing a resorption rate comparable to the bone formation one [17,18]. 

This behavior of CS particles was confirmed by the present study: When used in combination with NHA, CS rapidly resorbs and converts into a desmoplastic connective tissue, leaving space for blood vessels ingrowth, while NHA particles remain to guarantee volume maintenance (Figure 7). Even though on one hand this aspect of NHA could be nonideal for postextraction sockets’ healing, on the other hand it is of paramount importance to avoid socket walls collapsing, due to its long-term resorption rate.

Furthermore, NHA showed osteocunductive behavior, being surrounded by a layer of newly formed bone. These findings are in accordance with histomorphometric outcomes reported in a recent study by Stacchi et al. in 2017 [22]. This group showed that after six months of healing there was a suitable extension of the bonding site between the host bone and NHA particles. Graft residual fragments were easily distinguishable from the other elements of the regenerated tissue and appeared to be joined by bridges of newly formed bone [22].

These considerations on NHA and CS behavior could suggest that the combination of these biomaterials may influence the quality of the regenerated bone, in terms of quantity of marrow spaces and vessels. This aspect could be of importance in implant dentistry, allowing the implant dentist to ideally choose the quality of the regenerated bone by varying the biomaterials ratio.

However, further investigations on the histological and biomechanical behaviors of different ratios of resorbable and nonresorbable bone substitutes, used in combination, is necessary to determine their proper clinical use.

## 5. Conclusions

The results of this clinical trial indicated that both NHA and CS, used alone or in combination, in postextraction sockets led to the development of newly formed bone after five months of healing. In particular, the combination of biomaterials presented no statistically significant difference with the control group, thus showing a better behavior if compared to the same biomaterials used alone. In fact, both CS and NHA showed a significantly lower bone formation percentage then the control group. 

When NHA was used in combination with CS, a statistically significant less residual graft particles percentage was shown when compared with NHA alone, leading to a higher percentage of bone formation while maintaining its role as a scaffold material.

The clinical effects of this human trial include the chance of increasing the choices for the replacement of bone autografts, as they do not always represent a promising or suitable option, due to their nature. In addition, sintered NHA and CS combine the benefit of high-tech improvement with the safety of synthetic biomaterials, preventing the theoretical potential risks of xenograft implantation in patients.

## Figures and Tables

**Table 1:** Patient characteristics at baseline.

Characteristics	Quantity
Males	5
Females	5
Mean age (range)	47.47 (24–63)
Smokers	2
Non-smokers	8
Total sites	16

**Table 2:** Area of the analyzed segments (mm^2^).

Tissue Parameters Analyzed	Samples	Missing	Mean	Std. Dev.	Std. Error	Range	Max	Min	Median	25%	75%
Total area CS	4	1	24.62	12.58	7.26	25.16	37.2	12.04	24.62	12.04	37.2
Total area NHA	4	1	8.53	0.85	0.49	1.7	9.38	7.68	8.53	7.68	9.38
Total area CS+NHA	4	1	23.49	7.85	4.53	15.69	31.33	15.64	23.49	15.64	31.33
Total area Ctr	4	1	9.62	1.81	1.05	3.62	11.43	7.81	9.62	7.81	11.43
Bone CS	4	1	2.29	1.12	0.64	2.23	3.4	1.17	2.29	1.17	3.4
Bone NHA	4	1	1.48	0.47	0.27	0.94	1.95	1.01	1.48	1.01	1.95
Bone CS + NHA	4	1	13.34	2.66	1.54	5.32	16	10.68	13.34	10.68	16
Bone Ctr	4	1	7.5	0.6	0.35	1.2	8.1	6.9	7.5	6.9	8.1
Connective CS	4	1	5.71	3.25	1.88	6.5	8.96	2.46	5.71	2.46	8.96
Connective NHA	4	1	4.89	2.33	1.35	4.66	7.22	2.56	4.89	2.56	7.22
Connective CS + NHA	4	1	3.91	1.99	1.15	3.98	5.9	1.92	3.91	1.92	5.9
Connective Ctr	4	1	2.17	0.36	0.21	0.72	2.53	1.81	2.17	1.81	2.53
Biomaterial CS	4	1	0.17	0.14	0.08	0.25	0.25	0	0.25	0	0.25
Biomaterial NHA	4	1	1.87	0.12	0.07	0.24	1.99	1.75	1.87	1.75	1.99
Biomaterial CS + NHA	4	1	1.22	0.82	0.47	2.0	2.04	0.4	1.22	0.4	2.04
Biomaterial Ctr	-	-	-	-	-	-	-	-	-	-	-

**Table 3:** Histomorphometric results in percentage (%).

Tissue Parameters Analyzed	Samples	Missing	Mean	Std. Dev.	Std. Error	Range	Max	Min	Median	25%	75%
Bone CS	4	1	13.56	13.08	0.08	0.27	0.3	0.03	0.09	0.03	0.3
Bone NHA	4	1	17.84	7.32	0.04	0.14	0.25	0.11	0.17	0.11	0.25
Bone CS + NHA	4	1	58.72	8.77	0.05	0.18	0.69	0.51	0.57	0.51	0.69
Bone Ctr	4	1	80.68	21.8	0.13	0.44	1.04	0.6	0.78	0.6	1.04
Connective CS	4	1	33.25	35.75	0.21	0.64	0.74	0.1	0.19	0.1	0.74
Connective NHA	4	1	55.88	21.86	0.13	0.44	0.77	0.33	0.57	0.33	0.77
Connective CS + NHA	4	1	17.34	8.51	0.05	0.17	0.25	0.08	0.19	0.08	0.25
Connective Ctr	4	1	22.62	0.52	~0	0.01	0.23	0.22	0.23	0.22	0.23
Biomaterial CS	4	1	0.56	0.52	~0	0.01	0.01	0	0.01	0	0.01
Biomaterial NHA	4	1	21.97	0.79	~0	0.02	0.23	0.21	0.22	0.21	0.23
Biomaterial CS + NHA	4	1	47.54	20.13	0.01	0.04	0.07	0.03	0.52	0.03	0.07
Biomaterial Ctr	-	-	-	-	-	-	-	-	-	-	-
